# Expression of PHB2 in Rat Brain Cortex Following Traumatic Brain Injury

**DOI:** 10.3390/ijms15023299

**Published:** 2014-02-21

**Authors:** Ting Xu, Xinjuan Fan, Yuanyuan Tan, Ying Yue, Weijie Chen, Xingxing Gu

**Affiliations:** 1 The Center Laboratory of Huai’an First People’s Hospital Nanjing Medical University, Huai’an 223300, China; E-Mail: xuting-1988@163.com; 2 Jiangsu Province Key Laboratory of Neuroregeneration, Nantong University, Nantong 226001, China; E-Mails: yuanyuan.easy@163.com (Y.T.); yueying1500628@163.com (Y.Y.); guxingxing@hotmail.com (W.C.); 3 Affiliated Hospital of Nantong University, Nantong 226001, China; E-Mail: m18262712560@163.com

**Keywords:** traumatic brain injury, Prohibitin2, neuron, astrocyte, proliferation, apoptosis

## Abstract

Prohibitin2 (PHB2) is a ubiquitous, evolutionarily strongly conserved protein. It is one of the components of the prohibitin complex, which comprises two highly homologous subunits, PHB1 and PHB2. PHB2 is present in various cellular compartments including the nucleus and mitochondria. Recent studies have identified PHB2 as a multifunctional protein that controls cell proliferation, apoptosis, cristae morphogenesis and the functional integrity of mitochondria. However its distribution and function in the central nervous system (CNS) are not well understood. In this study, we examined PHB2 expression and cellular localization in rats after acute traumatic brain injury (TBI). Western Blot analysis showed PHB2 level was significantly enhanced at five days after injury compared to control, and then declined during the following days. The protein expression of *PHB2* was further analyzed by immunohistochemistry. In comparison to contralateral cerebral cortex, we observed a highly significant accumulation of PHB2 at the ipsilateral brain. Immunofluorescence double-labeling showed that PHB2 was co-expressed with NeuN, GFAP. Besides, PHB2 also colocalized with activated caspase-3 and PCNA. To further investigate the function of PHB2, primary cultured astrocytes and the neuronal cell line PC12 were employed to establish a proliferation model and an apoptosis model, respectively, to simulate the cell activity after TBI to a certain degree. Knocking down PHB2 by siRNA partly increased the apoptosis level of PC12 stimulated by H_2_O_2_. While the PHB2 was interrupted by siRNA, the proliferation level of primary cultured astrocytes was inhibited notably than that in the control group. Together with our data, we hypothesized that PHB2 might play an important role in CNS pathophysiology after TBI.

## 1. Introduction

Traumatic brain injury (TBI) is a most serious cause of injury related hospitalization, disability, and death throughout the world which can cause a significant loss of productive years and the necessity for long term care placing a large economic burden on society [1–4]. Despite exhaustive medical therapy, the prognosis for TBI patients remains poor due to the development of cerebral edema, elevated intracranial pressure, neuronal and vascular injury, and long-term cognitive dysfunction. The current medical therapies exhibit limited efficacy in reducing neurological injury and the prognosis for patients remains poor. Thus, an improved mechanistic understanding of the pathogenesis of TBI is needed to develop more efficacious strategies for intervention [5–7]. TBI is composed of a primary insult resulting from the direct result of the mechanical effects of impact and inertial forces, and a secondary insult which can induce a progressive cascade of related events in the brain damage and death of the TBI suffer that contribute to neuronal cell death, astrocyte proliferation, and microglia activation, including ischemia, brain edema, diffuse axonal injury, excitotoxicity, radical-mediated damage, and mitochondrial dysfunction [6–12].

Prohibitin2 (PHB2) is ubiquitous, evolutionarily conserved proteins that is present in multiple cellular compartments. It belongs to the prohibitin family, which comprises two subunits, PHB1 and PHB2 [13,14]. Initial investigations focused on the role of PHB1 as an inhibitor of cell proliferation hence the original name prohibitin [15]. Disrupting the PHB genes has effects ranging from decreased replicative lifespan in yeast to a larval arrest phenotype in the fruit fly [16]. PHB1 has a molecular mass of about 32 kD and PHB2 has a molecular mass of about 37 kD [17,18]. Together, these proteins can form a high molecular weight complex, which localized in mitochondria, the plasma membrane and the nucleus [19,20]. As many studies report, PHB1 is a potential tumor suppressor protein, which has been shown to inhibit cell proliferation and repress E2F transcriptional activity [21,22]. In addition, PHB2 can regulate cell life activity by controlling cleavage of OPA1, which is necessary for cristae morphogenesis of mitochondria [23–26]. Moreover PHB2 also acts as a transcriptional repressor for MAF2 and MyoD [27–29]. It has been reported by Hideaki and Sachihiro, that interfering in the expression of PHB2 in Hela cells can resulted in premature sister-chromatid separation and defects in chromosome congression accompanied by mitotic arrest by spindle-checkpoint activation [30]. PHB2 also can interact with HAX-1 to inhibit apoptosis and regulate the mitochondrial morphology [31–33]. When the brain suffers damage, a series of pathological and physiological reactions would be caused, including apoptosis, proliferation, and differentiation, especially in the region around the wound. In our previous study we found significantly elevated PHB2 expression in neurons and astrocytes after TBI. Current studies have confirmed that TBI can cause neuronal apoptosis as well as the activation and proliferation of astrocytes [4–6]. Therefore, we reasoned that PHB2 may participate in the physiological activity of the neuron and astrocyte following TBI. This study was done to gain greater insight into the functions of PHB2 in the adult CNS and its cellular and molecular mechanisms underlying central nerve lesion and repair.

## 2. Results and Discussion

### 2.1. The Expression of PHB2 in Brain Cortex by Western Blot after TBI

**Figure 1.**
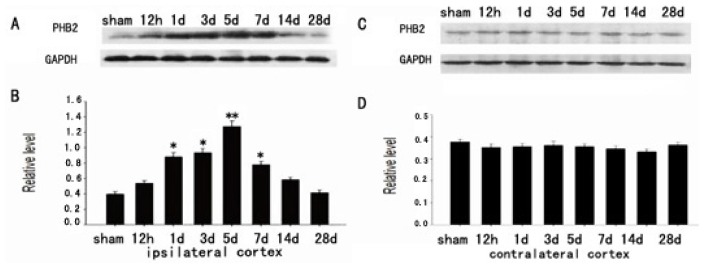
The expression of PHB2 in brain cortex by Western Blot during TBI. Western Blot was performed to confirm the temporal patterns of PHB2 expression during traumatic brain injury (TBI). In the cortex surrounding the wound, The PHB2 protein levels were relatively lower in the sham-operated brain cortex, then increased from 12 h after TBI, peaked at day five, and then gradually decreased to the normal level (**A**) while protein level had no alterations in the control group (**C**); Quantification graphs (relative optical density) of the intensity of staining of PHB2 to GAPDH at each time point (**B**,**D**). GAPDH was used to confirm equal amount of protein was run on gel. The data are means ± SEM, * *p* < 0.05, ** *p* < 0.01 (*n* = 5).

Western Blot assays were performed to confirm the temporal pattern of PHB2 expression during traumatic brain injury (TBI). In the cortex surrounding the wound, The PHB2 protein levels were relatively lower in the sham-operated brain cortex, then increased from 12 h after TBI, peaked at day five, and then gradually decreased to the normal level (Figure 1A,B), while there were no alterations in the control group (Figure 1C,D). These data indicated that PHB2 protein level had a temporal change after TBI.

### 2.2. The Staining Changes of PHB2 Immunoreactivity in Rat Brain Cortex after TBI

**Figure 2.**
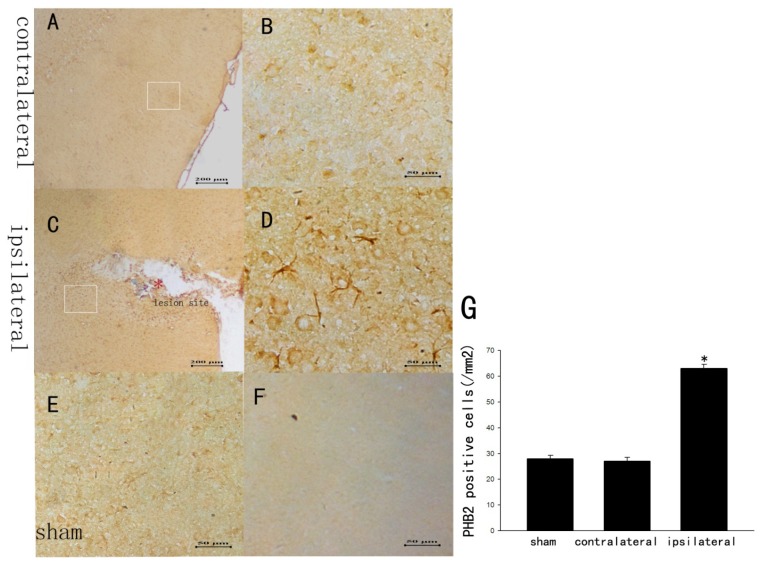
The staining changes of PHB2 immunoreactivity in rat brain cortex after TBI. We performed immunohistochemistry with anti-PHB2 rabbit polyclonal antibody on transverse cryosections of brain cortex and investigated the temporal pattern of PHB2. We could see that the immunostaining of PHB2 was widely distributed throughout the rat brain with a relatively lower level staining in the contralateral and sham brain, and the major cellular morphology of PHB2-positive cells appeared as neuron (**A**,**B**,**E**); While the expression pattern was different, that expression was intensive and the cellular morphology appeared as both neurons and astrocytes at day five after TBI (**C**,**D**); Quantitative analysis documented that there was a dramatic elevation of PHB2-positive cells after TBI (**G**); (**F**) Immunostaining of negative control for PHB2. Error bars represent SEM, Scale bars: 200 μm (**A**,**C**), 50 μm (**B**,**D**–**F**), * *p* < 0.05 (*n* = 3).

We could see that the immunostaining of PHB2 staining was widely distributed throughout the rat brain in the contralateral and sham brain but with a relatively lower level staining, and the major cellular morphology of PHB2-positive cells appeared as neuron (Figure 2A,B,E), while the expression pattern was different that expression was intensive and the cellular morphology appeared as both neurons and astrocytes at day 5 after TBI (Figure 2C,D). Quantitative analysis documented that there was a dramatic elevation of PHB2 positive cells after TBI (Figure 2G).

### 2.3. The Colocalization of PHB2 with Different Cellular Markers by Double Immunofluorescent Staining

**Figure 3.**
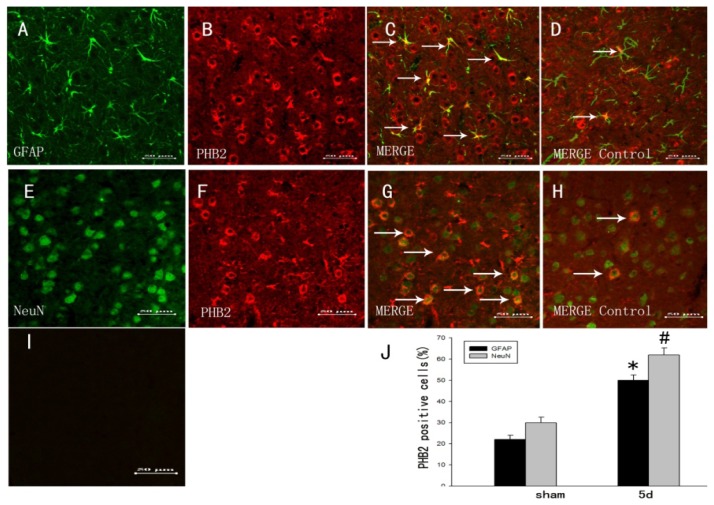
The colocalization of PHB2 with different cellular markers by double immunofluorescent staining. In the adult rat brain cortex within 1 mm distance from the lesion site at the fifth day after TBI, horizontal sections labeled with cell-specific markers: NeuN (a marker of neurons), and GFAP (a marker of astrocytes). We found that PHB2 was positive with astrocytes (**A**–**C**); Meanwhile, the positive PHB2 in neurons were also demonstrated by co-staining with anti-NeuN (**E**–**G**); To identify the proportion of each phenotype-specific marker-positive cells expressing PHB2, cell counting was performed in control and TBI five-day group. PHB2 expression was significantly higher in neurons and astrocytes (**C**,**G**) at five days compared with sham brain (Figure 3**D**,**H**); (**I**) Negative control. Quantitative analysis of NeuN and GFAP-positive cells expressing PHB2 (%) in the sham-operated group and day five after injury (**J**). Error bars represent SEM, Scale bars: 50 μm (**A**–**I**), * *p* < 0.05, *# p* < 0.05 (*n* = 3). The white arrows represent the co-expression of protein.

To further investigated the cell types expressing PHB2 after TBI, we used double immunofluorescent microscopy with two cell-specific markers: NeuN (a marker of neurons), and GFAP (a marker of astrocytes). We found that PHB2 was positive with astrocytes (Figure 3A–C). Meanwhile, the positive PHB2 in neurons were also demonstrated by co-staining with anti-NeuN (Figure 3E–G). To identify the proportion of each phenotype-specific marker-positive cells expressing PHB2, cell counting was performed in the control and TBI five-day group. PHB2 expression was significantly higher in neurons and astrocytes (Figure 3C,G) at five days compared with sham brain (Figure 3D,H).

### 2.4. Association of PHB2 with Survival and Proliferation after TBI

**Figure 4.**
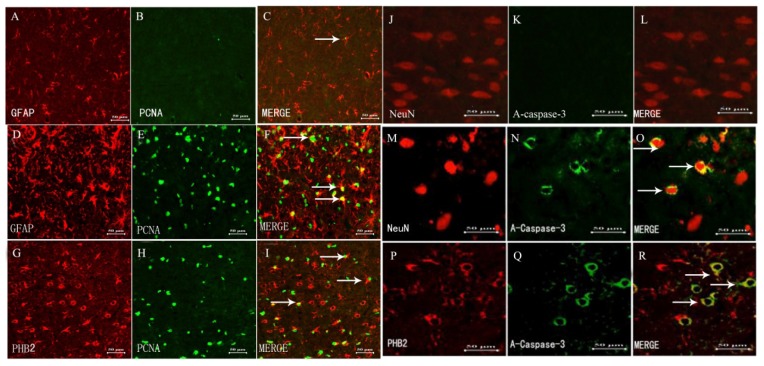
Association of PHB2 with survival and proliferation after TBI. Double immunofluorescence staining was used for PCNA, GFAP, activated caspase-3 and PHB2 in brain cortex after TBI. In adult rat brain at five days after injury or in the sham group, sections labeled with PCNA (**B**,**D**) and GFAP (**A**,**C**), and the co-localization of PCNA with GFAP were found in brain (**C**,**F**); PHB2 positive cells were co-localized with PCNA (**G**–**I**); Sections labeled with activated caspase-3 (**K**,**N**) and NeuN (**J**,**M**), and the co-localization of activated caspase-3 with NeuN were shown in the brain at day five after TBI (**O**); Moreover, coincidence of PHB2 (**P**) with activated caspase-3 (**Q**) were also showed (**R**) by adjacent serial sections. Scale bars: 50 μm. The white arrows represent the co-expression of protein.

To explore the potential role of PHB2 in survival and proliferation after TBI. Double immunofluorescence staining was used for PCNA, GFAP, activated caspase-3 and PHB2 in brain cortex after TBI. In the adult rat brain at five days after injury or the sham group, sections labeled with PCNA (Figure 4B,E) and GFAP (Figure 4A,D), and the co-localization of PCNA with GFAP were identified in brain (Figure 4C,F). The majority of reactive astrocytes were PCNA-positive at five days after TBI (Figure 4F) and the PCNA-positive astrocytes were increased in the experiment group compared to the sham group (Figure 4C). In the rat brain after TBI, PHB2-positive cells were also co-localized with PCNA (Figure 4G–I). Sections labeled with activated caspase-3 (Figure 4K,N) and NeuN (Figure 4J,M), and the co-localization of activated caspase-3 with NeuN was also shown in the brain after TBI. Moreover, there was also a coincidence of PHB2 (Figure 4P–R) with activated caspase-3 at five days after TBI by adjacent serial sections.

### 2.5. The Expression Change of PHB2 in the Process of the Proliferation of Astrocytes Stimulated by LPS

To further explore the role of PHB2 in the proliferation of astrocytes, we used LPS to stimulate the proliferation of primary astrocytes to mimic cell proliferation after TBI to a certain extent. A certain stimulation concentration of LPS (1 μg/mL) was added to the purified primary astrocytes. Cell protein was collected at 4, 6, 8, 10, 12, 24 h after stimulation. Western Blot assays were used to detect the expression level of PHB2 at each time point. Results showed that the expression of PHB2 was lower in the control group, and increased at 4 h after stimulation, then reached a peak at 10 h after stimulation then decreased. These results indicate that PHB2 might play a role in astrocyte proliferation stimulated by LPS.

### 2.6. The Influence of PHB2 for Proliferation of Primary Cultured Astrocytes Induced by LPS Confirmed by BrdU ELISA Detection Technology

**Figure 5.**
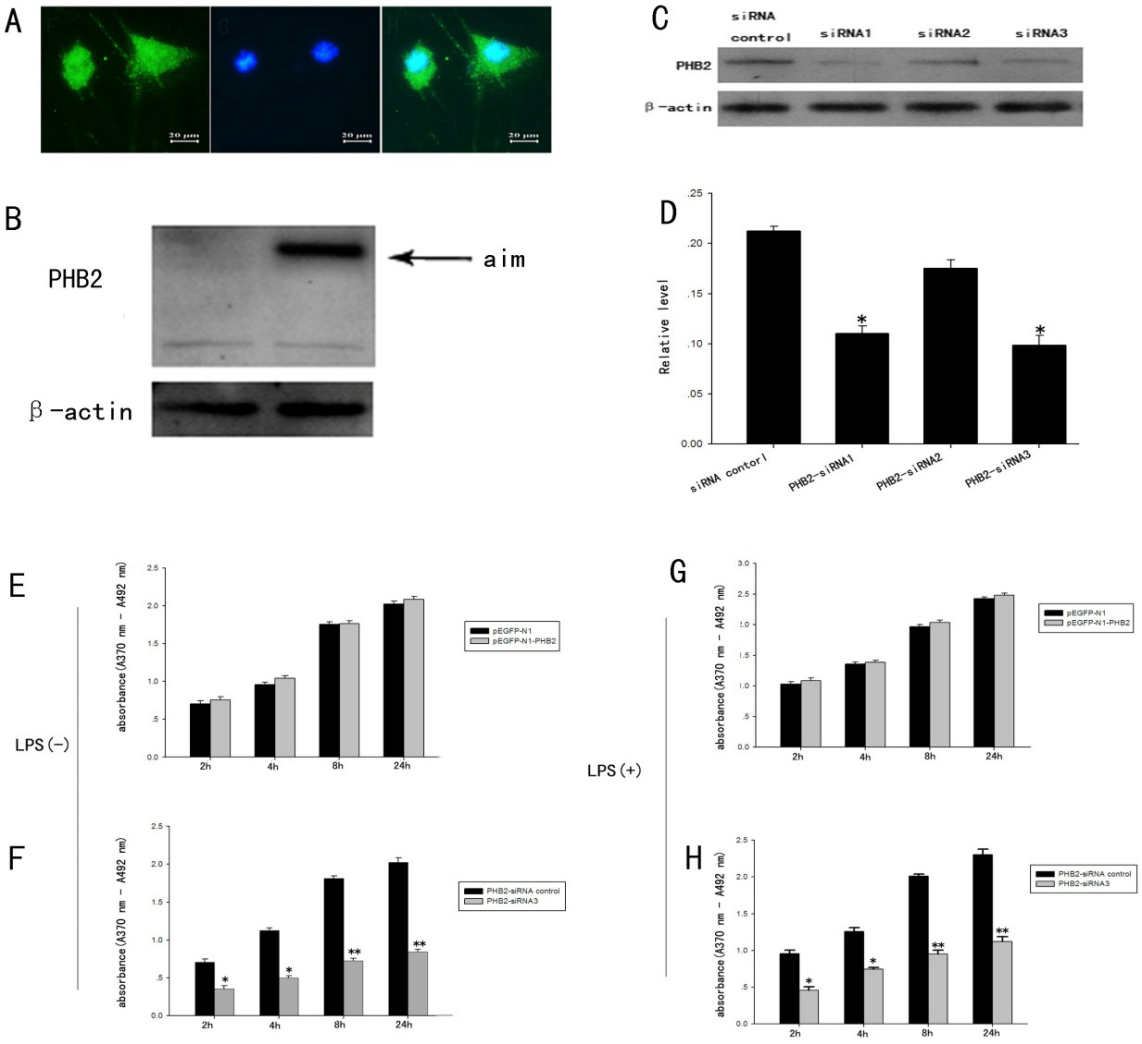
The influence of proliferation of primary cultured astrocytes induced by LPS confirmed by BrdU ELISA detection technology. Immunofluorescence showed that PHB2 localized in both cytoplasm and nucleus of primary cultured astrocytes (**A**); Western Blot results showed that, there was a band at the anticipated size (the size of fusion protein) position on the cellulose acetate membrane after transfection with pEGFP-N1-PHB2 (**B**); but not transfection with pEGFP-N1. As shown by Western Blot analysis, siRNA3 made a significant effect in reducing PHB2 expression (**C**,**D**); BrdU ELISA results show that proliferation of astrocytes, induced by LPS (**G**,**H**); was higher than that in the control group (**E**,**F**); (**E**,**G**) showed that the proliferation of astrocytes which over-expressed PHB2 was slightly higher than that in the control group, but there was no significant difference; (**F**,**H**) Cell proliferation was inhibited after the PHB2 was interfered with in astrocytes, resulted in a significant difference from the control group. Error bars represent SEM, Scale bars: 20 μm (**A**), * *p* < 0.05, ** *p* < 0.01 (*n* = 3).

Immunofluorescence showed that PHB2 localized in both the cytoplasm and nucleus of primary cultured astrocytes (Figure 5A). Evidence shows that PHB2 is expressed both in the cytoplasm and nucleus, and its distribution reflects different functions, such as to regulate cell activity by OPA1, act as transcription factor or as a regulator for mitosis. To further recognize the effect of PHB2 on astrocytes, we regulated its expression by over-expressed plasmid or siRNA to observe the influence of PHB2 for astrocytes activity. The fusion plasmid pEGFP-N1-PHB2 and empty plasmid were transfected into primary astrocytes by Lip2000 (Invitrogen, Carlsbad, CA, USA). Twenty-four hours after transfection, cells protein was collected. Western Blot results showed a band at the anticipated size position of the fusion protein on the cellulose acetate membrane after transfection with pEGFP-N1-PHB2 (Figure 5B) but not with pEGFP-N1. We constructed several PHB2 knockdown siRNAs. As shown by Western Blot analysis, siRNA3 had a significant effect in reducing PHB2 expression (Figure 5C,D).

We regulated the expression of PHB2 by transfection with an overexpression plasmid or siRNA of PHB2 into astrocytes, and applied BrdU ELISA detection technology to further explore the relationship between PHB2 with proliferation of astrocytes. After incubation with Brdu-POD for the respective times: 2, 4, 8, and 24 h, the ability of cell proliferation was analyzed by ELISA. BrdU ELISA results showed that proliferation of astrocytes, induced by LPS (Figure 5G,H), was higher than that in the control group (Figure 5E,F), indicating that LPS was successful in inducing astrocyte proliferation in this experiment. Moreover, results showed that the proliferation of astrocytes which were over-expressed PHB2 was slightly higher than that in the control group but with no significant difference (Figure 5G), while cell proliferation was inhibited after PHB2 was interfered with in astrocytes, resulted in a significant difference from control group (Figure 5H). These results suggest a latent relationship for PHB2 with the proliferation of astrocytes.

### 2.7. The Relationship between Astrocytes, Mitosis and PHB2

**Figure 6.**
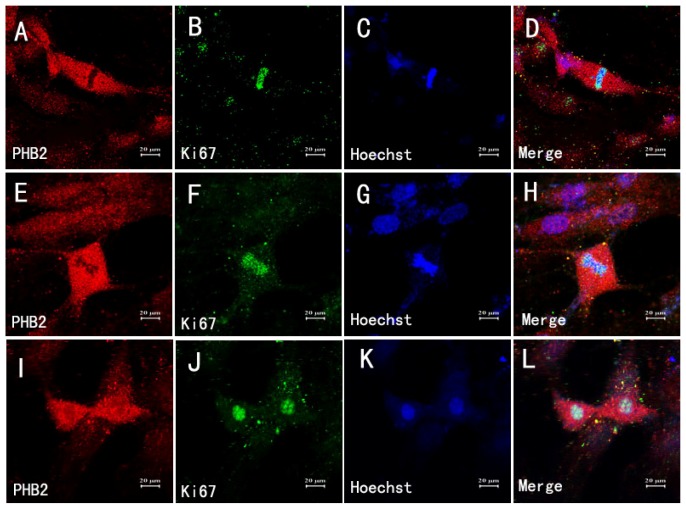
The relationship between astrocytes mitosis and PHB2. Fluorescent double labeling demonstrates: Cells at late metaphase showed high expression level state of PHB2 (**A**,**E**,**I**); PHB2 and Ki67 (marker of cell proliferation) (**B**,**F**,**J**) double-fluorescent staining results further prove these types of cells were in late metaphase (**D**,**H**,**L**). Hoechest in **C**,**G**,**K** is the marker of nuclear. Scale bars: 20 μm (**A**–**L**).

It has been reported that PHB2 is also connected to mitosis of Hela cells. Thus, we further explored the potential role of PHB in the process of astrocyte mitosis. Fluorescent double labeling showed (Figure 6): cells at late metaphase showed high expression level states of PHB2 (Figure 6A,E,I), and PHB2 and Ki67 (marker for proliferating cells) (Figure 6B,F,J) double fluorescent staining results further proved that these types of cells were in late metaphase, while other cells showed lower expression states of PHB2. These results suggest that PHB2 might have an important role for mitotic astrocytes.

### 2.8. The Expression Change of PHB2 in H_2_O_2_-Induced PC12 Cell Apoptosis

**Figure 7.**
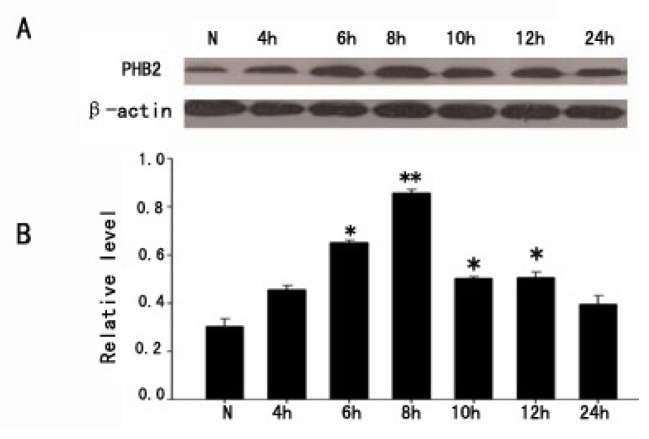
The expression change of PHB2 in H_2_O_2_-induced PC12 cells apoptosis. (**A**) The time courses of PHB2 expressions in PC12 cells after H_2_O_2_ stimulus; β-actin was used to confirm equal amounts of protein run on gel; (**B**) Quantification was made at each time point, the bar chart showed the ratio of PHB2 to β-actin at each time point; these data are means ± SEM. * *p* < 0.05, ** *p* < 0.01 (*n* = 3).

Previous studies showed PHB2 colocalization with active caspase-3 in neurons, thus we suspected PHB2 might take part in the process of neuronal apoptosis after TBI. We explored a H_2_O_2_-induced PC12 cell apoptosis model for subsequent research. A certain stimulation concentration of H_2_O_2_ (0.25 μM) was added to the PC12 cells. Cell protein was collected at 4, 6, 8, 10, 12, and 24 h after stimulation. Western Blot assays were used to detect the expression levels of PHB2 at each time point. Results showed that PHB2 increased in PC12 stimulated by H_2_O_2_, and peaked at 8 h (Figure 7A). These results indicate that PHB2 might play a role in apoptosis of PC12 cells stimulated by H_2_O_2_. However, further study is required to determine on whether it acts as protected or not.

### 2.9. The Potential Role of PHB2 during the Process of PC12 Apoptosis Induced by H_2_O_2_ Confirmed by Flow Cytometry-Based Annexin V-PE/7-AAD Staining Analysis

**Figure 8.**
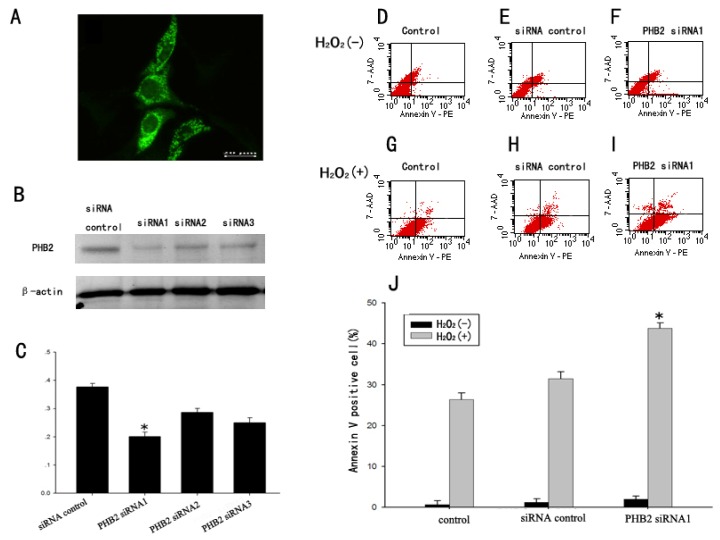
The potential role of PHB2 during the process of PC12 apoptosis induced by H_2_O_2_ confirmed by Flow cytometry-based Annexin V-PE/7-AAD staining analysis. Immunofluorescence was applied to identify the localization of PHB2 in PC12. Results showed that PHB2 was mainly localized in the cytoplasm of PC12 cells (**A**); Western Blot showed that siRNA1 had a significant effect in reducing PHB2 expression (**B**,**C**); (**D**–**I**) Flow-cytometry-based assays showed that PHB2 siRNA1 increased the number of apoptotic cells. In the four fields of the original images from the flow cytometry-based study, the number of the dots indicates the number of Annexin V-PE−/7AAD−(the bottom-left field), Annexin V-PE+/7AAD−(the bottom-right field), Annexin V-PE−/7AAD+ (the top-left field), and Annexin V-PE+/7AAD+ cells (the top-right field), respectively; (**J**) Quantifications of the results from the flow-cytometry-based study showed that PHB2 siRNA1 induced significant increases in the number of Annexin V-P+/7AAD− cells after H_2_O_2_ stimulation (**F**,**I**), while control did not (**D**,**G**) nor non-specific siRNA (**E**,**H**) show notable impact. Data were collected from three independent experiments. Error bars represent SEM, Scale bars: 20 μm (**A**), * *p* < 0.05 (*n* = 3).

Immunofluorescence was applied to identify the localization of PHB2 in PC12. Results showed that PHB2 was mainly localized in the cytoplasm of PC12 cells (Figure 8A). It has been demonstrated that PHB2 is expressed in both the nucleus and cytoplasm. The PHB2 in the cytoplasm can interact with cell apoptosis-regulated protein HAX-1 to protect cells from apoptosis. We proposed that PHB2 may play a protected role in PC12 apoptosis induced by H_2_O_2_. Similar to above, we transfected PC12 cells with siRNA to knockdown PHB2 expression. As shown by Western Blot analysis, siRNA1 made a significant effect in reducing PHB2 expression (Figure 8B,C).

Next, we used flow cytometry-based assays to determine cell apoptosis by assessing the levels of Annexin V-PE-positive cells. 7-AAD is impermanent to live cells and apoptotic cells, but stains dead necrosis cells with red fluorescence, binding tightly to the nucleic acids in the cell. We found that H_2_O_2_ stimulation led to significant increases in Annexin V-PE-positive cells, suggesting that the H_2_O_2_ induced neuron apoptosis cell model was well established (Figure 8D,G), and non-specific siRNA did not change the apoptotic cell percent significantly (Figure 8E,H). However, when PHB2 was knocked down, PC12 cells became more sensitive to H_2_O_2_ and induced apoptosis, as the apoptotic cell number increased notably (Figure 8F,I). Quantification analysis was illustrated in Figure 8J.

### 2.10. Discussion

TBI is a brain injury disease, caused by trauma. As falling and traffic accidents happen more frequently in recent years, TBI has become the most serious cause of disability and death in the 45 years old population. Moreover, because of high morbidity and mortality, TBI has become a serious disease that poses a threat to human health in current society. In addition to the major harm for human health, the loss of labor and the expensive cost for treatment caused by TBI can also bring a great burden for society and the economy [6,13]. It is important for us to recognize the pathogenesis of TBI to draw up effective therapeutic measures.

In this study, we establish a TBI model with a conditioned stereotactic knife lesion in adult rats to mimic clinically acute TBI, especially from sharp instrument injury of the brain. Western Blot analysis revealed that the protein level of PHB2 peaked at day five after TBI. Immunohistochemistry showed that PHB2 was widely distributed throughout the rat brain, and the major cellular morphology of PHB2-positive cells appeared as neurons in the normal brain, while the expression pattern after TBI was different in that cellular morphology appeared as both neurons and astrocytes. Immunofluorescence results further confirmed that PHB2 was colocalized with neurons and astrocytes, while PHB1 was abundantly expressed in neurons and oligodendroglia, but not in astroglia [34,35]. As recent studies reported, the complex of PHB1 and PHB2 is necessary for maintaining the function of mitochondria, but the respective functions of PHB1 and PHB2 for cell activity are diversely different and other functions responsible for the different cellular distributions are not well explored. In this experiment, the proportion of PHB2-positive cells was increased in the surrounding the areas of damage in neurons and astrocytes after TBI. To sum up, it appears that moderate traumatic brain injury may be associated with enhanced expression of PHB2 in the brain. In addition, the colocalization of PHB2/PCNA (proliferating cell nuclear antigen) or activated caspase-3 was detected in the injured brains. Collectively, these data were consistent with the hypothesis that PHB2 was implicated in the CNS pathophysiology after TBI.

TBI can cause a series of cellular and molecular events, evolving over a period of hours to several days, particularly in the area surrounding the lesion, such as glial proliferation and reactive. Astrocytes play a neuroprotective effect for their ability to take up potassium and glutamate and release mitogenic factors [36–38]. However, astrocytes retract their end feet from vessels, resulting in increased permeability, and as a result, give rise to a glial scar in response to injury [39,40]. Since both of these events are harmful for the brain, it is necessary and important for vascular leakage and subsequent brain damage limitation to learn how these complex cells respond to insult. In this study, immunofluorescence double-labeling shows that PHB2-positive astrocytes are rarely observed in the normal rat cortex but are enhanced around the lesion after injury. Meanwhile, our staining results indicated that the GFAP-positive astrocytes surrounding the lesion also expressing PCNA and PHB2 was colocalized with PCNA. For these reasons, we may suppose that PHB2 might play an important role in astrocyte proliferation after CNS injury.

**Figure 9.**
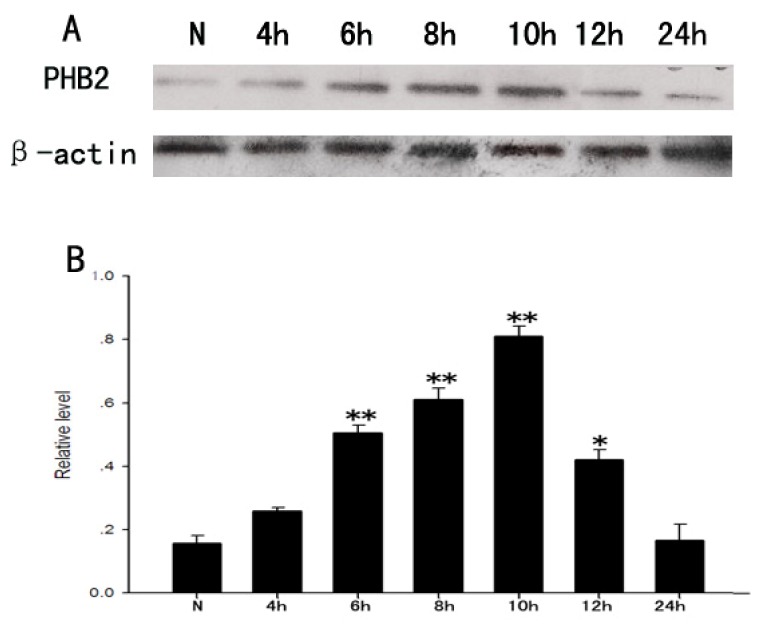
The expression change of PHB2 in the process of the proliferation of astrocytes stimulated by LPS. (**A**) The time courses of PHB2 expressions in astrocytes after LPS stimulus; β-actin was used to confirm equal amounts of protein was run on gel; (**B**) Quantification was made at each time point, the bar chart showed the ratio of PHB2 to β-actin at each time point; these data are means ± SEM, * *p* < 0.05, ** *p* < 0.01 (*n* = 3).

In the *in vitro* experiment, we used lipopolysaccharide (LPS) to stimulate proliferation of astrocytes. Western Blot detection of PHB2 protein levels in the Figure 9 showed that expression levels of PHB2 was increased at the stimulation point of 4 h, then began to decrease at 12 h but was still higher than normal level at 24 h after stimulation. These results indicate that a relatively higher expression of PHB2 was required for proliferation of astrocytes. In addition, we regulated the expression of PHB2 by transfection of overexpressed plasmid or siRNA of PHB2 into astrocytes, and applied BrdU ELISA detection technology to further explore the relationship between PHB2 with proliferation of astrocytes. BrdU test results showed that proliferation of astrocytes that over expressed PHB2 was slightly higher but not significantly different than that in the control group. However, cell proliferation was significantly inhibited after PHB2 was interfered with in astrocytes, resulting in a considerable difference from the control group. The above results show that interfering with expression of PHB2 significantly inhibited cell division suggesting a view that proves contrary to that proliferation of astrocytes requires relatively high expression levels of PHB2. However, what role does PHB2 play for astrocyte proliferation? Hideaki and Sachihiro reported that knockdown of PHB2 in Hela cells can cause abnormal separation of sister chromatids due to an abnormal agglutination of sister chromatids and order in the metaphase plate in the early stage of mitosis, thus affecting the process of cell progression from prophase to anaphase. Double immunofluorescence was performed to identify the relationship between PHB2 with Ki67. Results showed that astrocytes in the late stage of mitosis had a high PHB2 expression state, while other cells showed a low expression state. Therefore, we hypothesized that the function of PHB2 for astrocytes might be similar to that in the research of Hideaki and Sachihiro. In summary, overexpression of PHB2 did not significantly affect cell proliferation, while interfering with expression could make a significant difference for cell proliferation, and astrocytes in the mitotic state were strongly positive for PHB2 suggesting that PHB2 plays an important role in the astrocyte mitotic proliferation process. PHB1 and PHB2 subunits were demonstrated essential for cell proliferation, which is dependent on conserved protein machinery in the outer and inner membrane, including prohibitin interaction with OPA1. Expression of various mutant PHB2 variants in PHB2-deficient MEFs revealed a striking correlation between cell growth and mitochondrial targeting of PHB2: only those PHB2 variants that were correctly targeted to mitochondria were capable of maintaining cell proliferation [28]. Thus we believe that astrocyte proliferation accompanied with the increased expression of PHB2 after TBI might be due to the high expression of PHB2 necessary for the high proliferation level of astrocytes.

To further determine the function of PHB2 in neurons, we employed a H_2_O_2_-induced cell apoptosis model in differentiated PC12 cells. Western Blot results showed that the expression of PHB2 was upregulated, induced by H_2_O_2_, indicating a relationship between apoptosis and PHB2. Immunofluorescence results showed that PHB2 was mainly localized in the cytoplasm of PC12 cells. It has been demonstrated that PHB2 can be expressed in both the nucleus and cytoplasm [4,20]. Mitochondrial PHB2, located in the cytoplasm, is involved in maintaining the morphology and function of mitochondria, and is closely related to mitochondrial-related cell activities, as well as interacting with cell apoptosis-regulated protein HAX-1, which can regulate mitochondrial function and protect cells against apoptosis [31–33]. Thus, we designed a PHB2 interference RNA to inhibit the expression of PHB2 in PC12 cells. Flow cytometry assays results showed that PC12 cells, in which PHB2 was interfered, were more sensitive to apoptosis induced by H_2_0_2_. Previous tissue immunofluorescence results showed that PHB2 was co-localized with activated caspase-3 in brain tissue after TBI, indicating a relation between PHB2 and cell apoptosis, and when expression of PHB2 was interfered with in PC12 cells, cells were more susceptible to apoptosis stimulation. All of the above results suggested that PHB2 may play a protective effect on PC12 cells and that the increased expression of PHB2 in neurons after TBI may be due to a feedback mechanism of nerve protection antagonistic to apoptosis. Chowdhury indicated that over-expression of PHB in undifferentiated GCs inhibit apoptosis which concomitantly results in an increased level of the anti-apoptotic proteins Bcl2 and Bclxl, reduced release of cytochrome c from mitochondria and inhibition of caspase-3 activity. In contrast, silencing of PHB expression resulted in change of mitochondrial morphology from the regular reticular network to a fragmented form, which enhanced sensitization of these GCs to the induction of apoptosis [41]. Merkwirth revealed prohibitin-deficient MEFs did not undergo apoptosis, but exhibited an increased susceptibility towards various stimuli of apoptosis [23]. In the adult brain, we observed PHB2 expression was not in all neurons and positive cells were significantly higher at five days compared with sham after brain injury. Because all the studies about prohibitin emphasized its essentiality in cell homeostasis [13–16], it seems difficult to explain the different PHB2 expression states in all neurons. Although feedback mechanisms of nerve protection seems reasonable, with the protective effect of PHB2 mediated through HAX-1 or other signal pathways, they are not proven, and the exact mechanisms need further investigation.

TBI secondary injury mechanisms include complex biochemical and physiological processes. The most important events contributing toward the pathology of TBI are reactive astrogliosis and microglial activation, which are initiated by the primary insult and manifest over a period of hours to days. In this period, astrocyte and microglial proliferation, diffuse axonal injury, excitotoxicity, radical-mediated damage, and mitochondrial dysfunction occur [1–7]. Temporal patterns of PHB2 expression after traumatic brain injury increased from 12 h, peaked at day five, and then gradually decreased to a normal level. This parallel with TBI secondary injury procession hints at important protective effects of PHB2 in this course. Bayer and other scientists discovered that some natural products called flavaglines have protective effects through cooperation with prohibitin in traumatic brain injury animal models [42–44]. Our study provides a potential experimental and theoretical basis for further research into the function of PHB2 in TBI and therapies for clinical trials. We believe a pharmaceutical product such as flavaglines might be useful in the early period of TBI if available.

## 3. Experimental Section

### 3.1. Animals and Surgery

Experiments were performed in accordance with National Institutes of Health Guidelines for the Care and Use of Laboratory Animals; all animal protocols were approved by the Department of the Animal Center, Medical College of Nantong University. Male Sprague Dawley rats (*n* = 65) with an average body weight of 250 g (220 to 275 g) were used in this study. All animals were housed with equal daily periods of light and dark and free access to food and water. Traumatic brain injury (TBI) model was used as described previously [45,46]. Rats were given an overdose of chloral hydrate and sacrificed at different time points post-operatively (*n* = 5 for each time point). Rats were deeply anesthetized with chloral hydrate (10% solution) and surgery was performed under aseptic conditions. An antero-posterior surgical incision (5-mm-long, 3-mm-deep, and 1-mm-wide) was made by inserting a micro knife into the right cortex 3 mm lateral from the midline (*n* = 40). Sham-operated animals (*n* = 5) were anesthetized and surgically prepared, but did not receive brain injury. The overlying muscles and skin were closed in layers with 4.0 silk sutures (Ethicon, Somerville, NJ, USA) and staples, respectively, and the animals were allowed to recover on a 30 °C heating pad. Animals were killed at 12 h, 1 d, 3 d, 5 d, 7 d, 14 d, and 28 d after injury. Sham-operated rats (*n* = 5) were sacrificed at three days. All surgical interventions and postoperative animal care were carried out in accordance with the Guide for the Care and Use of Laboratory Animals (National Research Council, Washington, DC, USA, 1996) and were approved by the Chinese National Committee to the Use of Experimental Animals for Medical Purposes, Jiangsu Branch. All efforts were made to minimize the number of animals used and their suffering.

### 3.2. Western Blot Analysis

To obtain samples for Western Blots, brain tissue surrounding the wound (70–90 mg) as well as an equal part of the contralateral, un-operated cortex were dissected out and immediately frozen at −80 °C until use. To prepare lysates, frozen brain tissues were minced with eye scissors in ice. The samples were then homogenized in lysis buffer (1% NP-40, 50 mmol/L Tris, pH 7.5, 5 mmol/L EDTA, 1% SDS, 1% sodium deoxycholate, 1% Triton X-100, 1 mmol/L PMSF, 10 mg/mL aprotinin, and 1 mg/mL leupeptin) and clarified by centrifuging for 20 min in a micro centrifuge at 4 °C. After determination of its protein concentration with the Bradford assay (Bio-Rad, Hercules, CA, USA), the resulting supernatant (50 μg of protein) was subjected to SDS-polyacrylamide gel electrophoresis. The separated proteins were transferred to a polyvinylidine difluoridemembrane (Millipore, Boston, MA, USA) by a transfer apparatus at 350 mA for 2.5 h. The membrane was then blocked with 5% nonfat milk and incubated with primary antibody against PHB2 (Anti-Rabbit, 1:500; Santa Cruz, CA, USA) or GAPDH (Anti-Rabbit, 1:1000; Santa Cruz, CA, USA). After incubation with an anti-rabbit or anti-mouse horseradish peroxidase-conjugated secondary antibody, protein was visualized using an enhanced chemiluminescence system (ECL, Pierce Company, Rockford, IL, USA).

### 3.3. Sections and Double Immunofluorescent Staining

After defined survival times, rats were terminally anesthetized and perfused through the ascending aorta with saline, followed by 4% paraformaldehyde. After perfusion, the brains were removed and post-fixed in the same fixative for 3 h and then replaced with 20% sucrose for 2–3 days, following 30% sucrose for 2–3 days. After treatment with sucrose solutions, the tissues were embedded in O.T.C. compound. Then, 10-μm frozen cross-sections were prepared and examined. All sections were first blocked with 10% normal serum blocking solution species the same as the secondary antibody, containing 3% (*w*/*v*) bovine serum albumin (BSA) and 0.1% Triton X-100 and 0.05% Tween-20 2 h at RT in order to avoid unspecific staining. Then the sections were incubated with both rabbit polyclonal primary antibodies for anti-PHB2 (Santa Cruz Biotechnology, Santa Cruz, CA, USA) and different markers as follows: NeuN (neuron marker, Chemicon, Temecula, CA, USA), activated caspase-3 (Santa Cruz Biotechnology, Santa Cruz, CA, USA), PCNA (Santa Cruz Biotechnology, Santa Cruz, CA, USA), GFAP (astrocyte marker, 1:200; Sigma, San Francisco, CA, USA). Briefly, sections were incubated with both primary antibodies overnight at 4 °C, followed by a mixture of FITC- and TRITC-conjugated secondary antibodies for 2 h at 4 °C. The stained sections were examined with a Leica fluorescence microscope (Leica, Solms, Germany).

### 3.4. Immunohistochemistry

After the sections were prepared, they were kept in an oven at 37 °C for 30 min, and rinsed twice in 0.01 M PBS for 5 min. All the sections were blocked with 10% donkey serum with 0.1% Triton X-100 and 1% BSA for 2 h at room temperature and incubated overnight at 4 °C with anti-PHB2 antibody (Rabbit, Santa Cruz, CA, USA), followed by incubation in biotinylated secondary antibody (Vector Laboratories, Burlingame, CA, USA). Sections were rinsed again for 5 min (three times) and incubated in the complex avidin-biotin-peroxidase (ABC Kit, Vector Laboratories, Burlingame, CA, USA) for 40 min at 37 °C. Staining was visualized with diaminobenzidin (DAB, Vector Laboratories, Burlingame, CA, USA). After reactions, the sections were dehydrated, cleared, and coverslipped. Slides were examined at 109 or 409 magnifications on a light microscope (Leica, Solms, Germany). Cells with strong or moderate brown staining were counted as positive, cells and no staining were counted as negative, whereas cells with weak staining were scored separately.

### 3.5. Reverse Transcription (RT)-PCR, Plasmid Construction and Transfections

Total RNA samples were prepared using Trizol reagent. RT-PCR was carried out according to the manufacturer’s instructions. Briefly, 4 μg of RNA was used for the RT reaction and 2 μL of the cDNA product was used in the PCR mix along with a forward primer, 5′CCGCTCGAGATGGCC CAGAACTTAAAGGACCTA3′ and a reverse primer, 5′CCGGAATTCGCTTCTTACCCTTAAT GAGGCTGT3′. Primers for β-actin were used as an internal control. Samples were amplified for 30 cycles of 94 °C for 15 s, 60 °C for 30 s, and 68 °C for 3 min. The PCR products were analyzed on 1% agarose gels. PHB2 cDNA was amplified using PHB2 specific primers containing XHOI and ECORI restriction sites. The purified 897-bp fragment and the pEGFP-N1 vector were cut with XHOI and ECORI and ligated to yield the recombinant PHB expression plasmid, pEGFP-N1-PHB2. Purified plasmid DNA was transfected into astrocyte using Lipofectamine 2000 (Invitrogen, Carlsbad, CA, USA) according to the manufacturer’s instructions.

### 3.6. Cell Culture

PC-12 cells are a commonly used neural cell line from *Rattus norvegicus* adrenal pheochromocytoma (a sympathetic nervous system tumor). They have general characteristics of neuroendocrine cells and can be passaged, so they are widely used in neurophysiology and neuropharmacology study.

The PC12 cells were maintained in a humidified incubator (5% CO_2_) in DMEM culture medium supplemented with 10% fetal calf serum, 1% glutamine, penicillin (100 U/mL), and streptomycin (100 mg/mL).

Primary cortical astrocyte cultures were prepared from 3–4 day old Sprague-Dawley rat pups as described by McCarthy and de Vellis (1980) [47] in accordance with the Guide for the Care and Use of Laboratory Animals (NIH publication number 80–23) as approved by the University of Nantong animal care and use committee. The cell culture media (DMEM/F12 containing 10% FBS supplemented with 100 U/mL penicillin, 100 μg/mL streptomycin,) was replaced every alternate day and on the eighth day the cells were transferred to a shaking incubator at 37 °C (260 RPM) for 24 h under air-restricted conditions to remove oligodendrocytes and microglia. The astrocyte-enriched cell cultures were allowed to grow for another four days and then on day 12 the astrocyte cell cultures were divided onto 100 × 20 mm poly-l-lysine coated culture dishes at a density of 1.0 × 10^6^ cells/dish. Cells were cultured at 5% CO_2_, 37 °C.

### 3.7. PHB2 RNA Silencing

The following sequences for PHB2-siRNA were synthesized: PHB2-rat-1002(1) sense: 5′GCCUCAUUAAGGGUAAGAATT3′, antisense: 5′UUCUUACCCUUAAUGAGGCTT3′; PHB2-rat-294(2) sense: 5′GCGUACAACAGGACACAAUTT3′, antisense: 5′AUUGUGU CCUGUUGUACGCTT3′; PHB2-rat-970(3) sense: 5′GCAGGAUGAAAGCUUUACUTT3′, antisense: 5′AGUAAAGCUUUCAUCCUGCTT3′. The siRNAs were commercially synthesized (Genepharma, Shanghai, China). For controls, scrambled RNA oligonucleotides were used. For each well, 33.3 nM of each of the three oligos were transfected using Lipofectamine 2000 (Invitrogen, Carlsbad, CA, USA) according to the manufacturer’s instructions. After incubation for six h, the medium was replaced with DMEM containing 10% fetal bovine serum. Transfected cells were used for the subsequent experiments 48 h after transfection.

### 3.8. Cell Proliferation ELISA, BrdU

Cells are cultured in the presence of the respective test substances in a 96-well MP at 37 °C for a certain period of time (1–5 days, depending on the individual assay system); subsequently, BrdU is added to the cells and the cells are reincubated (usually 2–24 h). During this labeling period, the pyrimidine analogue BrdU is incorporated in place of thymidine into the DNA of proliferating cells; After removing the culture medium the cells are fixed and the DNA is denatured in one step by adding FixDenat (Roche, CH, Basel, Switzerland) (the denaturation of the DNA is necessary to improve the accessibility of the incorporated BrdU for detection by the antibody); The anti-BrdU-POD binds to the BrdU incorporated in newly synthesized, cellular DNA; the immune complexes are detected by the subsequent substrate reaction; and the reaction product is quantified by measuring the absorbance at the respective wavelength using a scanning multiwell spectrophotometer (Thermo Fisher Scientific, Waltham, MA, USA). The developed color and thereby the absorbance values directly correlate to the amount of DNA synthesis and thereby to the number of proliferating cells in the respective microcultures.

### 3.9. Flow Cytometry-Based Annexin V-PE/7-ADD Staining

PC12 cells were inoculated into six well plates, with transfection of PHB siRNA and control interference, 30% hydrogen peroxide was added to each well and incubated for 24 h, keeping the final stimulation concentration as 0.25 μM. Cells were stimulated for 8 h, and collected by EDTA without trypsin digestion (Note: trypsin digestion time should not go too long, otherwise it might easily lead to false positives). Cells were washed with PBS twice (2000 rpm, 5 min), and collected in (1~5) × 10^5^ cells. Then 500 μL of Binding Buffer was added for the cell suspension; ten microliter of labeled Annexin V-PE was added into 100 μL of the cell suspension. After a 15-min incubation on ice, 380 μL binding buffer and 10 μL 7AAD (7-amino-actinomycin D) solution were added into the cell suspension. Subsequently the number of stained cells was assessed by a flow cytometer (BD FACS AriaII, New York, NY, USA).

### 3.10. Quantitative Analysis

Cells double labeled for prohibitin and the other phenotypic markers used in the experiment were quantified. Sections were double labeled for PHB2 and NeuN, GFAP. To identify the proportion of each phenotype-specific marker-positive cells expressing PHB2, a minimum of 200 phenotype-specific marker-positive cells were counted 1 mm from the wound in each section. Then double-labeled cells for PHB2 and phenotype-specific markers were recorded 1 mm to the wound center in each section. Two or three intermittent sections (50 μm apart) per animal were sampled.

### 3.11. Statistical Analysis

All data were analyzed with Stata 7.0 statistical software (StataCorp, College Station, TX, USA). All values were expressed as mean ± SEM. One-way ANOVA followed by the Tukey’s post-hoc multiple comparison tests was used for statistical analysis. *p* values less than 0.05 were considered statistically significant. Each experiment consisted of at least three replicates per condition.

## 4. Conclusions

The expression of PHB2 was induced after TBI, and this change might play an important role in astrocyte proliferation and neuronal apoptosis after CNS injury.

## Abbreviations

CNS: Central nervous system
ECL: Enhanced chemiluminescence system
PAGE: Polyacrylamide gel electrophoresis
BSA: Bovine serum albumin
NeuN: Neuronal nuclei
GFAP: Glial fibrillary acidic protein
PCNA: Proliferating cell nuclear antigen
GAPDH: Glyceraldehyde-3-phosphate dehydrogenase
CASPASE-3: cysteinyl aspartate-specific protease 3
MEFs: mouse embryonic fibroblasts
